# POTENTIAL UTILITY OF FOVEAL MORPHOLOGY IN PRETERM INFANTS MEASURED USING HAND-HELD OPTICAL COHERENCE TOMOGRAPHY IN RETINOPATHY OF PREMATURITY SCREENING

**DOI:** 10.1097/IAE.0000000000002622

**Published:** 2019-07-24

**Authors:** Samira Anwar, Mintu Nath, Aarti Patel, Helena Lee, Samantha Brown, Irene Gottlob, Frank A. Proudlock

**Affiliations:** * Ulverscroft Eye Unit, Robert Kilpatrick Clinical Sciences Building Leicester Royal Infirmary, University of Leicester, Leicester, United Kingdom;; † Department of Ophthalmology, University Hospitals of Leicester NHS Trust, Leicester, United Kingdom;; ‡ Department of Cardiovascular Sciences, Glenfield Hospital, University of Leicester, Leicester, United Kingdom;; § Department of Ophthalmology, Clinical and Experimental Sciences, Faculty of Medicine, University of Southampton, Southampton University Hospital, Southampton, United Kingdom; and; ¶ Department of Neonatology, University Hospitals of Leicester NHS Trust, Leicester Royal Infirmary, Infirmary Square, Leicester, United Kingdom.

**Keywords:** foveal morphology, hand-held optical coherence tomography, prematurity, preterm infant

## Abstract

**Purpose:**

To investigate dynamic foveal morphology with postmenstrual age, in preterm infants with and without retinopathy of prematurity using hand-held optical coherence tomography, adjusting for gestational age (GA) and birthweight (BW).

**Methods:**

Prospective mixed cross-sectional/longitudinal observational study of 87 participants (23–36 weeks GA; n = 30 with, n = 57 without retinopathy of prematurity) using hand-held optical coherence tomography images (n = 278) acquired between 31 weeks and 44 weeks postmenstrual age excluding treated retinopathy of prematurity. Measurements included foveal width, area, depth, central foveal thickness, maximum slope, and parafoveal retinal thickness at 1,000 *µ*m nasal and temporal to the central fovea.

**Results:**

Retinopathy of prematurity was significantly correlated with only foveal width in either GA or BW adjusted statistical models. In contrast, severity of prematurity (GA, BW) correlated with foveal area (*P* < 0.005), depth (*P* ≤ 0.001), and slope (*P* < 0.01), although central foveal thickness (*P* = 0.007) and parafoveal retinal thickness (*P* < 0.001) correlated with GA, but not with BW.

**Conclusion:**

Foveal width is independent of GA and BW with potential in retinopathy of prematurity screening assessment using hand-held optical coherence tomography. Foveal morphology could be graded in prematurity during development, with possible implications for future management of preterm infants.

## Abstract 2

Supplemental Digital Content is Available in the Text.

Hand-held optical coherence tomography in prematurity shows foveal depth, thickness and area correlate with severity of gestational age or birthweight rather than presence of retinopathy of prematurity. In contrast, foveal width is independent of gestational age and birthweight but not retinopathy of prematurity, and has potential in retinopathy of prematurity screening.

Preterm birth is defined by gestational birth age (GA) before 37 weeks^1^ and is associated with very low birthweight (BW) (less than 1,500 g) or extreme low BW (less than 1,000 g).^2^ Infants born at less than 32 weeks GA and those with extreme low BW are vulnerable to retinopathy of prematurity (ROP), a potentially blinding disease that requires primary screening and intervention during the period of retinal development before 42 weeks to 44 weeks postmenstrual age (PMA). Determining the infants that benefit most from ROP intervention is based on retinal appearance and is mainly subjective, varying between specialists.^3^ Therefore, a key goal of ROP study has been to investigate factors that identify preterm infants at risk of treatment requiring ROP.

Hand-held optical coherence tomography (HH-OCT) is a noninvasive imaging technology permitting high resolution detail of the central retina to be rapidly acquired from infants at the earliest stages of prematurity. Studies are now emerging that use HH-OCT to chart foveal changes in prematurity, for example, to compare the effect of treatments for ROP.^4^ Earlier HH-OCT studies in preterm infants report persistence of inner retinal layers across the foveal depression, increased thickness of the inner retina, shallow foveae, and reduced depth.^5–8^ However, because GA, BW, and ROP are all strongly correlated, the relationship between foveal changes observed on OCT with severity of prematurity and changes associated with ROP remains unclear.^9^

Our aim was to investigate dynamic changes in foveal morphology parameters with PMA using HH-OCT to identify indicators that differentiate between diagnosis of ROP and non-ROP, which could not be accounted for by differences in GA or BW. To achieve this, we imaged preterm infants with and without ROP between 31- and 44-weeks PMA using HH-OCT. We excluded treated ROP infants and modelled the fovea using a difference of Gaussians (DoG) fit.

## Methods

The study was conducted in accordance with the tenets of the Declaration of Helsinki and granted approval by a Local Ethics Committee (NRES committee, Nottingham, East Midlands, United Kingdom). Patients were recruited from the Leicester Royal Infirmary neonatal and maternity unit, United Kingdom. All preterm babies from 31 weeks to 44 weeks PMA who required ROP screening were eligible for inclusion in the study. Abnormal ocular examinations other than diagnosis of ROP, and treated ROP were exclusion criteria. Data from infants requiring treatment were included up until treatment was performed.

Retinopathy of prematurity screening, staging, and treatment criteria of preterm infants were performed according to the United Kingdom guidelines.^10^ For the purpose of the study, ROP was defined as Stages 1 to 3 using the United Kingdom guidelines (Stages 4 or 5: partial or total retinal detachment, respectively, were excluded from the study; see appendix C of the United Kingdom ROP Guidelines^10^). Infant eyes were instilled with topical dilating drops (Cyclomydril = cyclopentolate hydrochloride 0.2% and phenylephrine hydrochloride 1%) and examined while awake, using a lid speculum. Binocular indirect ophthalmoscopy was used to establish the presence or absence of ROP. Documentation of demographic and clinical parameters for each preterm infant included: PMA, GA, and BW, presence or absence of ROP, the stage of ROP if present, single or multiple birth, sex, eye (right or left), and ethnicity (Caucasian or non-Caucasian). The number of infants who switched from no ROP to ROP and vice versa where ROP regressed spontaneously during the course of imaging, was also documented.

### Scan Acquisition and Selection of Foveal B-Scan

Imaging was performed from 31 weeks to 44 weeks PMA at 1 to 2 weekly intervals. Optical coherence tomography scanning was conducted in both eyes using a portable noncontact high-resolution HH-OCT (Envisu C-Class; Leica Microsystems, Wetzlar, Germany). Scans were optimized for obtaining a single high-quality scan at the central retina consisting of 500 A scans and 100 B scans, covering a rectangular volume 5.0 mm × 10.0 × 2 mm. The total scan time was 2.9 seconds (5.8 milliseconds per B scan). The lateral distance settings (defined for adults on the machine) were corrected to account for the smaller axial lengths in the infant population using a conversion table according to PMA and GA from the data presented by Maldonado et al.^11^

We aimed to acquire five HH-OCT images per infant per eye. From the HH-OCT scans acquired for each infant, those with the brightest and clearest components on retinal scanning were chosen for analysis. The successful identification of the foveal center was achieved by examining five uninterrupted B scans on either side of the B scan with the deepest point in the central retina.^12^ A foveal depression could always be identified on inspection. Repeated longitudinal images from each infant from 31 weeks to 44 weeks PMA were included in the study and analysis.

### Image Segmentation and Foveal Contour Measurement

The fovea was modelled using a DoG customized fit based on previous literature^13,14^ and analysis of the images was performed using customized layer segmentation macros written in ImageJ software (United States National Institutes of Health, Bethesda, MD, https://imagej.nih.gov/ij/, downloaded on December 2013). Foveal parameters included width, area and depth, central foveal thickness (CFT), steepest slope of the foveal wall, and parafoveal retinal thickness (pRT).

Foveal B-scans were flattened using the Bruch membrane as a reference line and translating individual A-scans vertically. Boundary detection of the internal limiting membrane (ILM) was performed automatically using the ABSnake plugin (http://imagejdocu.tudor.lu/doku.php?id=plugin:segmentation:active_contour:start downloaded on December 2013). Manual fine adjustment of the fitted line was used to generate the final segmentation.

Foveal shape dimensions were analyzed using an enhanced model based on the DoG principle described by Dubis et al.^13^ Because the fovea is asymmetric as reported by Liu et al,^14^ we modelled the nasal and temporal aspects of the fovea separately.

The DoG fits were calculated using Solver, an add-in tool in Excel (Microsoft Corporation, Seattle, WA). The aim of the Solver Tool was to reduce the root sum of squares of the differences between the actual and fitted values by adjusting the height and width terms of the Gaussians. An additional term was added to reduce the error between the bottom of the foveal pit values and the nasal and temporal fit. The starting points approximate to typical foveal profile consisting of a narrower inverted Gaussian which mainly fits the pit and a wider noninverted Gaussian which mainly fits the parafovea.

In the models by Dubis et al^13^ and Liu et al^14^ the two rim points that determine the maximum diameter of the foveal depression were taken to be at the highest points on the two sides of the pit. This was determined from points of inflection, where the direction of the ILM changes direction and the slope is zero. However, in many preterm infant images, the foveal contour continues to increase beyond the foveal rim and the wider Gaussian fitting of the parafovea can also follow an inverted profile in contrast to adult retina. Hence, in our model, the foveal rim edge was defined as the maximum point of the third derivative of the ILM profile, which is the earliest indication of the falling away of the ILM to form the foveal pit. For consistency, this method was used on all images including those where the parafovea was fitted with a noninverted Gaussian.

**Fig. 1.**
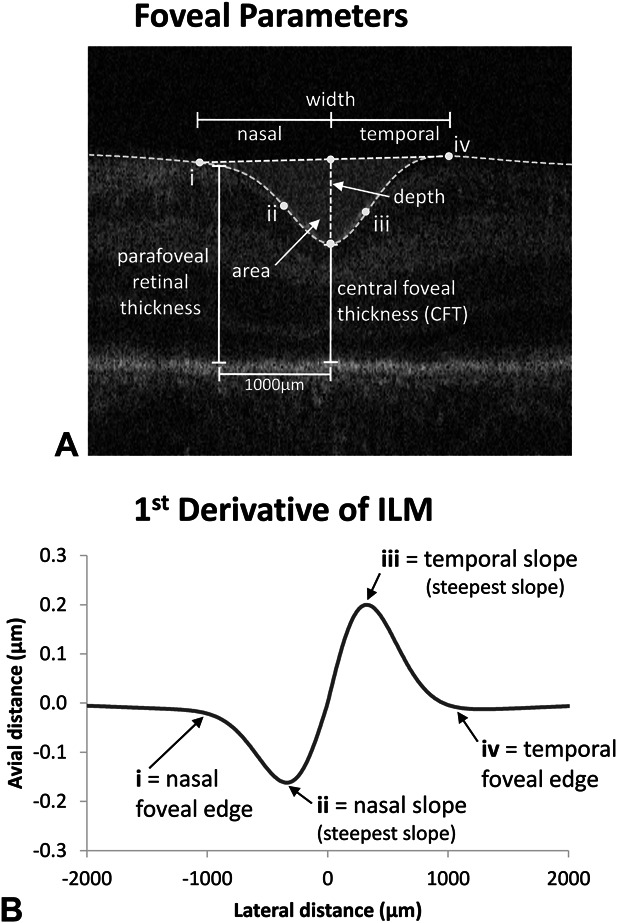
**A.** Foveal parameters are shown with respect to an original optical coherence tomography B-scan image. The nasal and temporal edges of the foveal depression are indicated by (i) and (iv), and the steepest foveal slope by (ii) and (iii), respectively. Parameters (i–iv) are defined using first, second, and third derivatives of the ILM. These are shown in (**B**) with respect to the first derivative of the ILM.

Figure 1A illustrates the foveal metrics of width, depth, area, and CFT in relation to the ILM profile. Figure 1B shows the first derivative of the ILM and points that used to define the rim edges and maximum nasal and temporal foveal slope. Nasal and temporal parameters of slope, width, area, and pRT (1000 *µ*m from the fovea) are illustrated in Figure 1A.

### Statistical Analysis

Multivariate mixed models were generated (see **Supplemental Digital Content 1**, http://links.lww.com/IAE/B43) to investigate the effect of diagnosis of ROP on foveal parameters described above with PMA, adjusting for the degree of prematurity (GA and BW) and other potential factors that may influence foveal morphology: ethnicity, birth (single/multiple) sex, and eye (right, left).^15–18^ Foveal asymmetry was also explored by comparing nasal and temporal measures of steepest slope of the foveal wall and pRT.

## Results

One hundred and seventy-four preterm infants were recruited to the study over 42 months (91 boys and 83 girls). Poor quality images for both eyes were discarded with the result that data could not be analyzed for 62 participants (36%) (31 boys and 31 girls). A further 25 infants (14%) developed cystic appearances (identical to cystoid macular edema) of the central retina^5,19–23^ distorting foveal structure, and these infants were also excluded from the foveal morphology analysis. The remaining 87 participants (47 boys and 40 girls) and 278 images were analyzed in the study. Fifty-seven infants (65%) never had ROP at any imaging session, whereas 19 infants (22%) had ROP recorded at every imaging session and 11 infants (13%) had ROP on at least one imaging session. In this last group (mixed ROP/no ROP), seven infants developed ROP in one eye, whereas one infant developed ROP in both eyes. In two infants, the ROP regressed spontaneously and another infant initially had no ROP recorded, which then developed into ROP and then subsequently spontaneously regressed. Details of the infant cohort are shown in Table 1. Further summary details of 1) number of successfully analyzed repeated images; 2) details of ethnicity, multiplicity, and sex; and 3) characteristics according to BW and GA are shown (see **Supplemental Digital Contents 2–4**, http://links.lww.com/IAE/B44, http://links.lww.com/IAE/B45, and http://links.lww.com/IAE/B46, respectively).

**Table 1.**
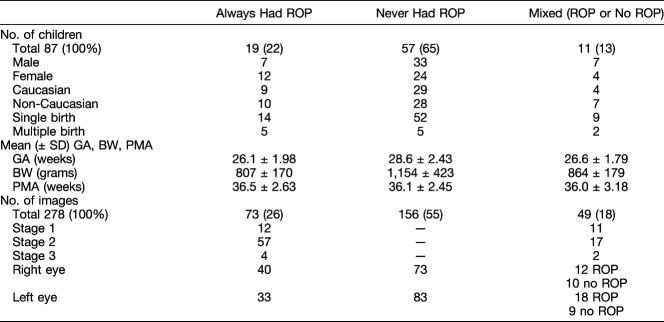
Participant and Image Characteristics

**Fig. 2.**
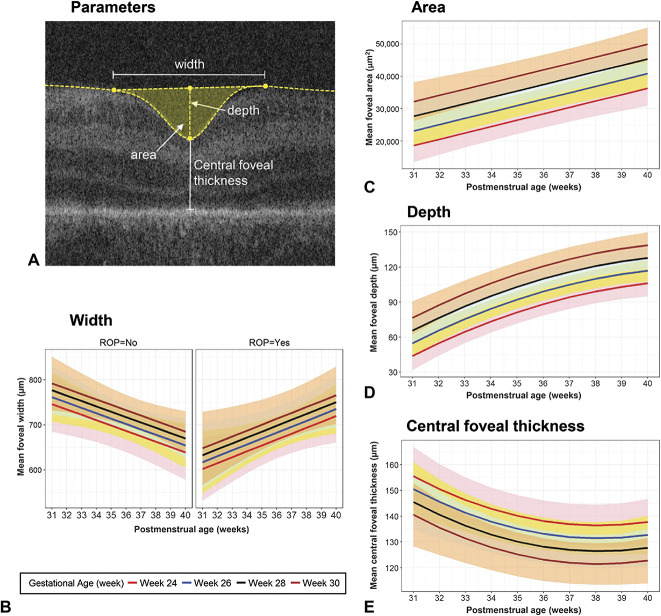
Foveal parameters (**A**), predicted mean fits (with 95% confidence intervals) of statistical models adjusted for GA for change with PMA: (**B**) foveal width, (**C**) area, (**D**) depth, and (**E**) CFT. Predicted mean fits are displayed for GA 24, 26, 28, and 30 weeks illustrated using colored lines, with matching shaded regions representing 95% confidence intervals of the mean. Absence/presence of ROP was only significant for foveal width, and hence plots for these two conditions are displayed in (**B**).

**Fig. 3.**
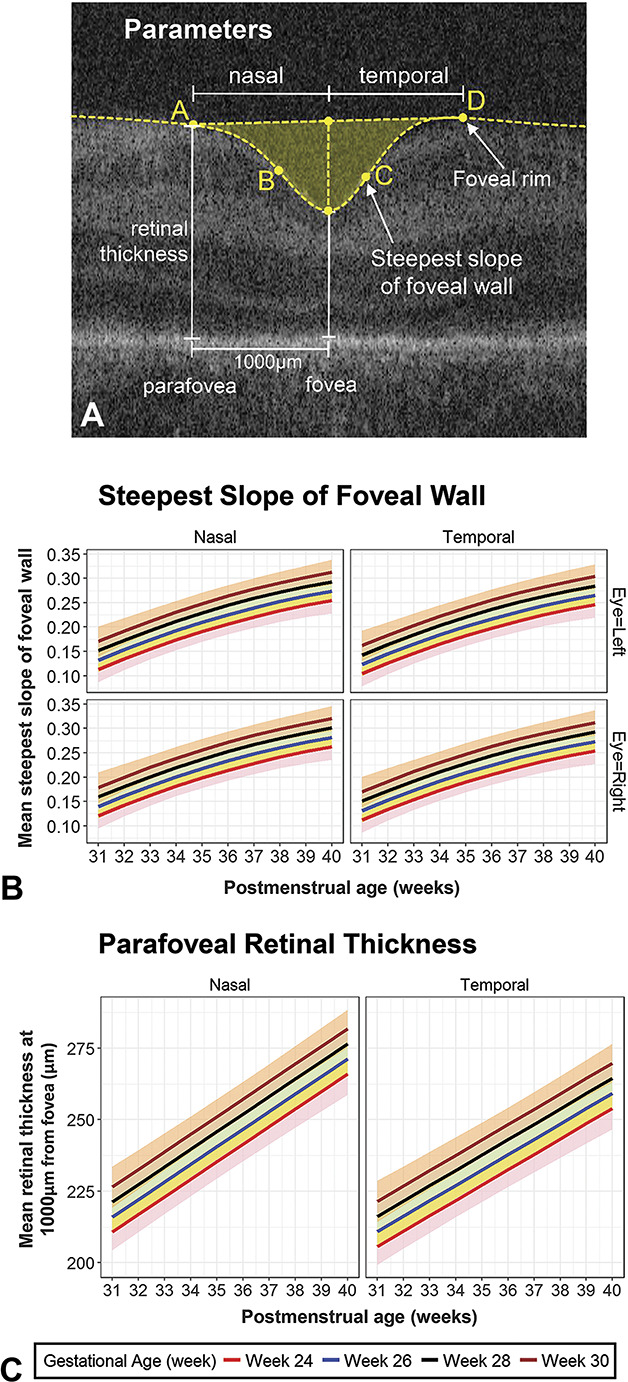
Foveal parameters (**A**) predicted mean fits (with 95% confidence intervals) of statistical models adjusted for GA for change with PMA, (**B**) steepest slope of the foveal wall, and (**C**) pRT. The predicted effect of GA on the mean difference in the parameter is illustrated using colored lines for GA 24, 26, 28, and 30 weeks, with matching shaded regions representing 95% confidence intervals of the mean. There was a significant difference for temporal and nasal aspects for both steepest slope and pRT. Eye (right or left) was also a significant factor for steepest slope.

**Fig. 4.**
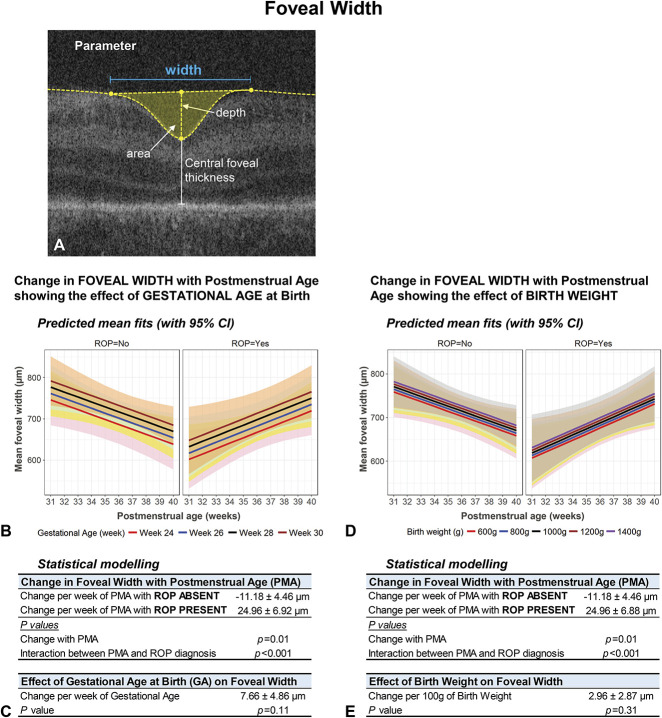
Foveal width (**A**, predicted mean fits with 95% confidence intervals) and results of statistical models of change in foveal width with PMA, adjusted for GA and BW. In (**B**) the predicted effect of GA on the mean difference in the foveal width is illustrated using colored lines for GAs 24, 26, 28, and 30 weeks, with matching shaded regions representing 95% confidence intervals of the mean. Similarly, this is shown in (**D**) for BW 600, 800, 1,000, 1,200, and 1,400 g. Results of the statistical models are shown in (**C**) and (**E**), respectively. Absence/presence of ROP had a significant effect on foveal width, and hence plots for these two conditions are displayed in (**B**) and (**D**).

Figure 2 shows predicted mean fits (with 95% confidence intervals) of statistical models adjusted for GA for: 1) foveal width (Figure 2B), 2) area (Figure 2C), 3) depth (Figure 2D), and 4) CFT (Figure 2E), and Figure 3 for: 5) steepest slope of the foveal wall (Figure 3B) and 6) pRT (Figure 3C). Separate plots are provided where statistical models demonstrate a factor that significantly affects the foveal parameter (e.g., presence or absence of ROP for foveal width, Figure 2B).

### Differences Between Retinopathy of Prematurity and Non-Retinopathy of Prematurity Infants

Figure 4 (see also Figure 2B) shows the results of multivariate modelling for GA and BW on foveal width, with predicted mean fits (with 95% confidence intervals) shown in Figure 4, B and D, respectively, and results of statistical modelling shown in Figure 4, C and E, respectively. Similar formats are used for foveal area (see **Supplemental Digital Content 5**, http://links.lww.com/IAE/B47), foveal depth (see **Supplemental Digital Content 6**, http://links.lww.com/IAE/B48), CFT (see **Supplemental Digital Content 7**, http://links.lww.com/IAE/B49), steepest slope of foveal wall (slope) (see **Supplemental Digital Content 8**, http://links.lww.com/IAE/B50) and pRT (see **Supplemental Digital Content 9**, http://links.lww.com/IAE/B51), respectively.

Foveal width was the only parameter where diagnosis of ROP had a significant effect, with a highly significant interaction between absence/presence of ROP and PMA (*P* < 0.001). Foveal width decreased when ROP was absent, at a mean (±SEM) rate of −11.18 ± 4.46 *µ*m per week, but increased when ROP was present at a rate of +24.96 ± 6.92 *µ*m per week (Figure 4, B and C). This interaction was independent of GA and BW, neither of which were correlated with foveal width (*P* = 0.11, *P* = 0.30). Exceptions to this trend were observed in 14.3% of infants without ROP (where foveal width increased ≥5% per week) and 18.2% of infants with ROP (where foveal width decreased ≥5% per week). For the three infants where ROP regressed, one infant showed an increase in foveal width (i.e., ≥5% per week between 32 and 37 weeks PMA), whereas two infants remained the same (<5% change per week).

The difference in trajectories with ROP absent and present resulted in a significant difference for the earliest PMA. On average, when ROP was present, foveal width was 76% of the value when ROP was absent at 32 weeks PMA (mean ± SE: 1,203.6 ± 84.40 *µ*m compared with 1,584.9 ± 77.25 *µ*m, respectively).

The results for the other foveal parameters as described above are shown (see **Supplemental Digital Contents 5–9**, http://links.lww.com/IAE/B47, http://links.lww.com/IAE/B48, http://links.lww.com/IAE/B49, http://links.lww.com/IAE/B50, and http://links.lww.com/IAE/B51, respectively) (see also Figures 2 and 3 to directly compare changes in parameters with PMA). In contrast to foveal width, absence or presence of ROP had no statistically significant effect on other parameters.

#### Parameters significantly correlated with gestational age and birthweight

Both GA and BW were significantly correlated with increasing foveal area (*P* < 0.001, *P* = 0.004) (see **Supplemental Digital Content 5**, http://links.lww.com/IAE/B47), depth (*P* < 0.001, *P* = 0.001) (see **Supplemental Digital Content 6**, http://links.lww.com/IAE/B48), slope (*P* < 0.001, *P* = 009) (see **Supplemental Digital Content 8**, http://links.lww.com/IAE/B50), and pRT (*P* < 0.001, *P* = 0.013) (see **Supplemental Digital Content 9**, http://links.lww.com/IAE/B51). However, only GA was a significant predictor for CFT (*P* < 0.001) (see **Supplemental Digital Content 7**, http://links.lww.com/IAE/B49), which decreased with increasing GA.

#### Other variables

Sex, ethnicity, and birth (single/multiple) were not significant predictors for any parameter in either model using GA or BW. The steepest foveal slope was greater in the right eye compared with the left eye (*P* < 0.01) (see **Supplemental Digital Content 8**, http://links.lww.com/IAE/B50). The nasal aspect of the fovea was significantly steeper (*P* = 0.001) and pRT significantly thicker (*P* < 0.001) compared with the temporal aspect (see **Supplemental Digital Contents 8 and 9**, http://links.lww.com/IAE/B50 and http://links.lww.com/IAE/B51) respectively.

#### Significant changes with postmenstrual age

All foveal parameters had significant dynamic mean rates of change with increasing PMA using both predictive models (*P* ≤ 0.01). Foveal depth (see **Supplemental Digital Content 6**, http://links.lww.com/IAE/B48), CFT (see **Supplemental Digital Content 7**, http://links.lww.com/IAE/B49), and steepest slope (see **Supplemental Digital Content 8**, http://links.lww.com/IAE/B50) all demonstrated significant nonlinear changes with PMA that were modelled with a quadratic term. The change in parameters with PMA are shown dynamically in the video animation (see **Supplemental Digital Content 10**, http://links.lww.com/IAE/B52) illustrating the change in the DoG model fits of ILM with increasing PMA for ROP and non-ROP groups. Particularly noteworthy is reducing foveal width in the group without ROP, which is not apparent in the group with ROP.

#### Parafoveal retina

An inverted Gaussian fitted the parafovea more often in the ROP group (75.7%) compared with the non-ROP group (55.5%) (chi-square test: *P* = 0.001). This can be observed in the video animation (see **Supplemental Digital Content** 10, http://links.lww.com/IAE/B69), where the parafovea slopes in more toward the fovea in the ROP group, especially at early PMAs.

## Discussion

We show that foveal width demonstrates a different trajectory of development depending on the presence or absence of ROP/non-ROP that is independent of GA and BW, factors that are clearly associated with the degree of prematurity. This is evident from a highly significant interaction between presence of ROP and PMA (*P* < 0.001), because of foveal width increasing in the ROP group and decreasing in the non-ROP group. Other parameters of foveal morphology show marked changes with PMA, but no differences exist depending on the presence or absence of ROP when models are adjusted for GA and BW.

Yanni et al^24^ studied older ex-preterm children (5–16 years of age), including ROP, and found shallow, less steep foveae, but no significant difference in foveal diameter compared with full-term born control children. However, their study had 4 preterm children with no ROP and included 15 children who had received treatment for ROP.

In contrast, our investigation of preterm infants shows a difference in foveal width between ROP and non-ROP, which is more apparent at early PMA. At 32 weeks, foveal width in the ROP group is 76% of the width in the non-ROP group. After 32 weeks, foveal width increases in the ROP group with increasing PMA, but decreases in the non-ROP group. Since this difference is found particularly in early PMA, foveal width may potentially differentiate between preterm infants that do not need further screening for ROP from those that do.

### Foveal Width as a Potential Early Indicator of Retinopathy of Prematurity

Risk algorithms to identify treatment requiring ROP (Type 1 ROP) are based on BW, GA, and weight gain as predictive variables in multivariate logistic regression models.^25^ The prospective PINT ROP study^26^ investigated such a model in extreme low BW infants; however, one infant with severe ROP, but not requiring treatment was missed. The authors highlight that factors associated with ROP in univariate analysis were not significant in multivariate analysis underlining the multifactorial nature of the risk in prematurity. The e-ROP study^27^ developed a model based on GA, weight gain, respiratory support data, and analysis of color image findings to predict ROP requiring treatment, producing risk scores. The results showed that image criteria predicted treatment requiring ROP better than GA and that this was best at 34 weeks PMA or earlier. In our study, we analyzed image characteristics using HH-OCT on foveal morphology and GA, BW, PMA, and identified foveal width as an early predictor variable independent of GA and BW. This suggests that HH-OCT of the fovea is a promising method that could be used with risk models using GA, BW, weight gain, and color image findings.

### Foveal Width, Foveal Avascular Zone and Retinopathy of Prematurity

It has been suggested that differences in the foveal width of older children and adults with a history of ROP could be related to the size of the foveal avascular zone (FAZ).^28^ The FAZ is determined by an absence of vessels in the macula, and has been correlated with foveal shape, continuation of the inner nuclear layer at the fovea, and increased foveal thickness.^24,29–31^ Chui et al^31^ studied 11 healthy adults and found that a smaller FAZ was associated with a thicker, narrower fovea. A small FAZ has also been noted in children aged between the ages of 1 year and 17 years with a history of prematurity.^32^ Falavarjani et al^33^ compared the FAZ in 15 preterm children (including those with ROP) with 11 age-matched controls between the ages of 4 years and 12 years using OCT angiography. They showed an abnormal FAZ in preterm children born less than 29 weeks GA. We found a significant correlation between earlier GA and increased CFT which is not influenced by diagnosis of ROP, suggesting that prematurity could result in the development of a smaller FAZ independently of the diagnosis of ROP. Future studies of the inner retinal layers at the FAZ using OCT during early active development of ROP may provide more information to explain the differences we found in foveal width between preterm infants with and without ROP.

### Foveal Parameters Dependant on Gestational Age and Birthweight

Previous studies in infants and ex-preterm children including ROP, report increased CFT with ROP, which is in contrast to our study, where CFT was independent of ROP. However, these conclusions are based on studies comparing preterm with full-term children,^19^ small numbers of non-ROP children,^8^ treated preterm children with ROP,^21^ or retrospective data.^23,34,35^

Our results on GA and CFT in preterm infants are in keeping with previous reports of older ex-preterm children that describe an association between CFT with GA, but not with diagnosis of ROP.^36–40^ Tariq et al^39^ showed that both GA and BW were significant predictors for increased foveal retinal thickness. Similarly, Bowl et al^40^ reported a cross-sectional analysis of RT at the foveal center and found inverse correlation between GA and BW with total retinal thickness in preterm children with and without ROP compared with full-term born children aged between 6 years and 13 years. However, by using separate predictor models, we found only a relationship for GA and not for BW, which is in contrast with these large studies of older preterm children. This may reflect either a change in foveal thickness between preterm birth and foveal maturity in childhood or differences in sample size.

Our finding of a greater early GA effect on CFT supports that of Wang et al^38^ who suggested that before 28 weeks GA, there is an increased likelihood of delayed migration of the inner retina away from the foveal center with persistence of the inner retina and increased CFT. A recent investigation by Molnar et al^41^ reported a strong association between central macular thickness and GA before 27 weeks in preterm children, after adjusting for ROP and sex. We also found that GA interacts with foveal depth similar to Rosen et al,^42^ who investigated foveal depth in preterm children aged 6.5 years including ROP. The correlation of increased CFT and reduced foveal depth with early GA in both preterm infants and older former preterm individuals suggests that extreme preterm birth interferes with the normal mechanisms of inner centrifugal retina migration at the fovea.

### Differences due to Sex and Ethnicity

Adults with normal foveae show differences between race and sex,^43^ male gender being associated with increased central macular thickness in preterm children.^41^ Our results present no differences in foveal dimensions between Caucasians and non-Caucasians, multiple/single birth infants, or sex, possibly because changes are not present in very early foveal development or because of insufficient numbers to reach significance.

## Limitations

Limitations of this study include conducting our study using one horizontal scan through the central fovea without analysis of the entire volume of the fovea. Also, we did not incorporate specific systemic confounders in our analysis such as oxygen therapy or illness with each individual, and it is known that these may relate to the severity and development of ROP. To adjust for the variability between the ROP and non-ROP groups for systemic factors, a larger number of participants would be needed to adjust for each disease category and oxygen delivery method.

A number of infants included in this study (n = 19) were only successfully scanned on one visit, although 78% (n = 68) were imaged ≥2 times and 49% (n = 43) infants were imaged ≥3 times. To investigate changes with time more systematically, it would be useful to develop a more consistent repeated scanning protocol for future studies.

We also did not incorporate FAZ measurements from fluorescein angiography in our investigation, because we were primarily concerned with modelling foveal morphology using OCT. The advent of portable OCT angiography in vivo would further our understanding of the relationship between ROP, the FAZ, and the foveal development.

## Conclusions

Foveal width in early PMA appears to have a significant relationship with ROP when adjusting for GA and BW. Further study may determine whether this has the potential to predict Type 1 ROP during screening using HH-OCT. The finding that only GA significantly influences CFT, supports the view that early birth interferes with inner retinal migration at the fovea, despite continuing development of the fovea. The EPICure@19 Study^44^ has reported a correlation between increased retinal thickness with a reduction in best-corrected visual acuity in adults born extremely preterm. The best-corrected visual acuity reduction was found to be similar in untreated ROP and non-ROP, suggesting that prematurity and not presence of ROP per se, has an impact on retinal thickness and vision.

A longitudinal HH-OCT study grading foveal morphology, GA, and visual acuity could be useful in understanding the changes that occur during visual development and in the management of children who are born preterm.
